# Perceptions of Heat-Susceptibility in Older Persons: Barriers to Adaptation

**DOI:** 10.3390/ijerph8124714

**Published:** 2011-12-19

**Authors:** Alana Hansen, Peng Bi, Monika Nitschke, Dino Pisaniello, Jonathan Newbury, Alison Kitson

**Affiliations:** 1 Discipline of Public Health, The University of Adelaide, Level 9, 10 Pulteney Street, Mail Drop DX650 207, Adelaide, SA 5005, Australia; Email: alana.hansen@adelaide.edu.au (A.H.); dino.pisaniello@adelaide.edu.au (D.P.); 2 South Australian Department of Health, PO Box 6, Rundle Mall, Adelaide, SA 5000, Australia; Email: monika.nitschke@health.sa.gov.au; 3 Spencer Gulf Rural Health School, University of South Australia, Box 3200, Port Lincoln, SA 5606, Australia; Email: jonathan.newbury@unisa.edu.au; 4 School of Nursing, Eleanor Harrald Building, Royal Adelaide Hospital, The University of Adelaide, Adelaide, SA 5005, Australia; Email: alison.kitson@adelaide.edu.au

**Keywords:** heat, elderly, vulnerability, public health

## Abstract

The increase in the frequency of very hot weather that is a predicted consequence of climate change poses an emerging threat to public health. Extreme heat can be harmful to the health of older persons who are known to be amongst the most vulnerable in the community. This study aimed to investigate factors influencing the ability of older persons to adapt to hot conditions, and barriers to adaptation. A qualitative study was conducted in Adelaide, Australia, involving focus groups and interviews with stakeholders including key personnel involved in aged care, community services, government sectors, emergency services and policy making. Findings revealed a broad range of factors that underpin the heat-susceptibility of the aged. These were categorized into four broad themes relating to: physiology and an age-related decline in health; socioeconomic factors, particularly those influencing air conditioning use; psychological issues including fears and anxieties about extreme heat; and adaptive strategies that could be identified as both enablers and barriers. As a consequence, the ability and willingness to undertake behavior change during heatwaves can therefore be affected in older persons. Additionally, understanding the control panels on modern air conditioners can present challenges for the aged. Improving heat-health knowledge and addressing the social and economic concerns of the older population will assist in minimizing heat-related morbidity and mortality in a warming climate.

## 1. Introduction

Extreme heat is a well documented threat to health, and the loss of lives associated with heatwaves exceeds that for any other natural hazard in Australia [1]. Unlike the more visual disasters such as bushfires and floods however, heat acts “silently” and the public are often unable to identify heatwaves as potential disasters that could claim lives [2]. It is well known from international and Australian reports that older persons are amongst the most vulnerable during heatwaves, with associations between heat and mortality being highest for the elderly, particularly those aged 75 years and over [3,4,5,6].

Impairments in the ability to regulate body temperature associated with age and disease have been described [7], but are not the sole reason for lowered heat tolerance in the aged. Adaptive capacity may be modified due to a number of underlying reasons. In very hot conditions for example, young healthy adults often cool down by simply turning on an air conditioner, wearing lightweight casual clothes, or consuming water. By contrast, older persons living independently in the community face certain barriers to behavior change and may be less likely to use these cooling options. 

Adelaide, South Australia, with a population of 1.2 million people [8], has a temperate climate with generally warm dry summers and regular heatwaves. In 2008 however, there was a heat event of record duration (fifteen consecutive days with maximum temperatures above 35 °C) [9]. This was followed in early 2009 by another extreme episode when temperatures exceeded 35 °C on nine consecutive days, six of which were above 40 °C, and the warmest ever overnight minimum temperature for the city was recorded [10]. The protection of public health during heatwaves became an issue of major importance as morbidity in the elderly increased [11]. Following the two heat events, new multilevel interventions were put in place including heat-health warnings and promotion via the media of heat-protective behaviors and neighbor awareness. During the events, agencies regularly contacted registered vulnerable and elderly clients to check on their wellbeing during the heat [12]. A five stage Extreme Heat Communications Plan for the State was subsequently formulated with objectives to communicate warnings regarding the potential impact of extreme heat, and provide advice on preparedness and preventive measures. Community involvement and awareness of those most at risk including the elderly became an integral part of the Plan [13]. 

With predictions of more frequent and intense bouts of hot weather as a consequence of climate change [14], it is important to focus on evidence-based strategies to minimize preventable heat-associated health events among the vulnerable aging population. The aim of this study was therefore to capture stakeholders’ views and experiences of recent extreme heat events in order to identify reasons for heat-susceptibility in older persons and perceived adaptation barriers during heatwaves. It was intended that findings would inform a subsequent study involving a telephone survey of persons aged 65 years or over. 

## 2. Methods

This qualitative study involved focus groups and interviews with persons associated with heat health management plans and/or the care of older persons. A thematic analysis, based on the research questions, was used to identify patterns within the data. Thematic analysis is an interpretative and flexible method of identifying themes in textual data. It is commonly used in public health research [15,16] and is highly suited to analyzing and reporting experiences of participants. Braun and Clarke (2006) provide comprehensive guidelines for using the methodology, detailing the steps involved together with the advantages and disadvantages of its use as a research tool to summarize key issues and find “repeated patterns of meaning” in qualitative data [16]. 

Ethics approval for the study was granted by the University of Adelaide Human Research Ethics Committee (No. H-035-2010), and the SA Health Human Research Ethics Committee (No. 362/04/2013).

### 2.1. Sampling

A key informant sample of stakeholders was selected consisting of persons with professional experience and expertise in dealing with the elderly, emergency management, or policymaking. *A priori* knowledge and recommendations from members of the research team and their associates assisted in the selection process. Those invited to participate included: medical practitioners; managers and front line staff in aged-care services; senior managers from emergency services and non-government organizations; executives from rural hospitals; and policymakers or personnel from several state government and local government departments in geographically and socio-economically diverse areas of the city of Adelaide. 

Potential respondents were approached either by an introductory telephone call or email in the first instance. On occasions, this resulted in another person in an organization being nominated and the process was repeated with the secondary contact. Information was provided about the study and an invitation to participate in either a focus group or face-to face interview was offered. Assurances of anonymity and confidentiality were provided. 

### 2.2. Data Collection and Analysis

Data collection was conducted between May and October 2010. There were 35 stakeholder respondents in total, as shown in Table 1. Four focus groups were held with between three and eight participants from a similar vocation in each. One focus group was held in a government department meeting room, one at an aged care facility, and two were held in a meeting room at The University of Adelaide. Respondents for whom a focus group interview was not convenient were interviewed face-to face at their workplace. One interview was conducted by telephone at the respondent’s request. 

Informed consent was provided by all respondents before interviews proceeded. Participants were given an interview topic guide with questions including: “In your experience why do some elderly folk become affected by the heat”; “In your opinion are there factors which make some more vulnerable than others and why”; “Compared to younger people are there particular challenges and barriers the elderly face in keeping cool and hydrated?” and “Did you find the situation more serious for the elderly during the January-February 2009 heatwave than in other heat events?”. Respondents were encouraged to elaborate on points of interest requiring clarification. They were also asked if they thought the research would be of value to their organization, and invited to make suggestions regarding the format of a forthcoming telephone survey of older people to be undertaken in two Australian states. Questions relating to the Extreme Heat Communications Plan and rural versus metropolitan issues were also asked but will not be discussed here. The same questions were asked of all participants whether in focus groups or face-to-face interviews. Focus groups were generally of one hour in duration; interviews lasted between 17 and 53 minutes, the longest being a group interview with two respondents. Proceedings were digitally recorded, transcribed verbatim into text and de-identified to assure confidentiality. 

**Table 1:** Respondent categories.

	Respondents	Females	Males
***Focus Groups***			
(1)	Policymakers, scientific officer	3	-
(2)	Policymakers, social worker, local government officer	4	-
(3)	Senior management (emergency services)	-	3
(4)	Aged care sector personnel	7	1
***Interviews***			
Senior policymakers		2	1
Medical practitioners (geriatrician, intensive care)		-	2
Manager (health services)		1	-
Executive officers (rural hospitals)		2	-
Local government officers/community workers		1	1
Senior emergency services officers		-	2
Manager (aged care sector)		1	-
Senior management (non-government organizations)		4	-
**Total**		**25**	**10**

Electronic versions of the data were imported into the qualitative analysis software package NVivo 8 (QSR International Pty Ltd., Doncaster, Australia). Data were analyzed using thematic analysis, an iterative process involving familiarization with the data, coding, collating of codes into themes, and reviewing the themes generated [16]. This firstly involved listening to the audio files and careful line by line reading and re-reading of the printed interview transcripts. Coding, which involves identifying passages of text that display certain ideas, themes or concepts, was undertaken using a bottom-up approach [17]. Notes were made by hand in the margins of the transcripts [15,16] and recurring concepts were identified in the printed and electronic versions of the data. ‘Free nodes’ were created in NVivo as themes emerged [17]. Conceptually similar segments of text were highlighted and copied into nodes that were given a name relevant to the theme. Segments of text were assigned to more than one node if required. Hyperlinks back to the transcripts and audio files allowed cross checking of data [17]. Nodes of a similar nature were collectively grouped into ’tree nodes’ in NVivo. Refining and reviewing of categories followed, and sub-categories or overarching categories were generated as necessary [16,18]. Original audio files were replayed to ensure themes were appropriate and no relevant points were overlooked. 

## 3. Results

The analysis revealed four broad analytical themes (1) physiological issues; (2) socioeconomic issues; (3) psychological issues; (4) adaptive strategies (barriers and enablers). The codes which comprised these themes are shown in Table 2. With the complex array of issues identified, separation of codes into these distinct categories was often subjective as issues were often interlinked and overlapping. The themes are discussed in detail below.

### 3.1. Physiological Issues

Stakeholders identified several physiological reasons for increased heat susceptibility in older individuals and discussed factors which rendered some more at risk than others (Table 2). Narratives revealed that compared to younger people, seniors often demonstrate a lower level of general health, reduced renal concentrating abilities, reduced sweat output and a diminished perception of heat. Complex medical issues, comorbidities, chronic conditions and genetic influences were also noted to contribute to homeostatic instability.

“*I think underlying chronic conditions is a big factor … around older people's tolerance for heat. In addition I think probably the second most significant factor is cognitive function.*”

(Manager, Non-Government Organization)

It was said that frailty and physical debility can impair the ability to take preventive action or access fluids, and mental and cognitive impairments can affect clarity of thought and adaptive behaviors. Dementia patients, particularly those pre-diagnosis, were said to be amongst the most vulnerable, and overdressing was seen as a problem for these individuals during hot weather. 

Insufficient fluid intake was seen as a significant problem during hot weather. It was noted that older people are often not well hydrated, and with a diminished thirst sensation, some simply forget to drink. Even if thirsty, many prefer hot tea to water, and it was generally thought they were “*not from a water-drinking era*” (Director, Health Services). Furthermore, it was noted that many are reluctant to increase their fluid intake for reasons including incontinence issues, use of diuretic medications or advice about fluid restrictions. 

Stakeholders with medical or nursing backgrounds discussed medications that can predispose users to heat stress and combinations of medications which can be problematic. These include medications that may interfere with renal function or sodium balance; cardiovascular active drugs that may impact on thermoregulatory mechanisms, and psychoactive medications which may cause confusion, sedation, or dull the senses. 

Rapid onset of extreme heat was said to be an issue as people did not have time to acclimatize. With lengthy heatwaves and warm nights providing little opportunity for respite, heat was noted to have a “*cumulative effect in a way*” (Director, Health Services) and people “*start to get a bit fatigued*” (Manager, NGO) over time and were generally less able to cope by the third consecutive day. This point was supported by the emergency services stakeholders who said there is often an upswing in workload by day three or four of a heatwave.

**Table 2:** Identified codes assigned to the four main themes.

Physiological Issues	Socioeconomic Issues	Psychological Issues	Adaptive Strategies	
			Barriers	Enablers
Thermophysiology	Cost of using A/C *	Anxieties raised during heat	Problems with A/C * use	Adaptive behaviors
Cognitive disorders	Financial concerns	Fears	Overdressing	Cooling centers
Comorbidities	Housing issues	Focus on heat	Don’t want to be a bother	Neighbors
Insufficient fluid intake	Isolation	Health misconceptions	Cultural issues	Resilience
Heat-related illness	Living alone	Life experiences	Housebound	Support networks
Medications	Socioeconomic status	Not busy	Don’t want to leave animals	
Mobility issues		Resistance to change	Heatwave length	
Hospitalization		Routines	Don’t swim	
Acclimatization		Security issues	Power outages	
Weight issues			Transport issues	
			Lack of health knowledge	

* A/C = air conditioning.

### 3.2. Socioeconomic Issues

Table 2 shows several socioeconomic and psychosocial barriers that were identified. Commonly discussed was the reluctance of older people in South Australia to use cooling devices in their homes due to the associated power costs. Replacing non-repairable older style air conditioners may not be within the financial capacity of some, and many did not have an air conditioner at all, according to respondents. Stakeholders spoke of the older generation as being frugal and proud, budgeting weekly so they can pay their bills on time and as a consequence, utility costs were said to be of major concern to many. One community worker said this was particularly so for those whose pensions had reduced from double to a single, whilst costs remained much the same. In referring to recent heatwaves, one stakeholder said:

“*Some people refused to turn their air conditioners on because of the cost. They said they couldn't afford it.*”

(Officer, Local Government)

Living arrangements were said to be important and that those who had support from family or neighbors fared better during periods of extreme heat, than those without. Social isolation, noted to be common amongst older people, was said to contribute to vulnerability as does isolation due to mental illness, dementia or illiteracy. During the severe 2009 heatwave, multilevel interventions were made by government and non-government organizations and several agencies made regular telephone contact with registered vulnerable and elderly clients to check on their wellbeing. Stakeholders noted however, that the most vulnerable in the community may be unlisted on databases due to isolation and not having someone to register them for services. 

### 3.3. Psychological Issues

Narratives revealed that extreme heat can cause distress to older persons. Those dealing with the elderly used terms such as “*overwhelming*”, “*panic*”, “*anxious*”, “*concern*”, “*worried*”, “*desperate*” and “*fear*” to describe emotional responses to extreme heat. Stakeholders indicated that during the intense 2009 heatwave, water restrictions, bushfires and extensive media coverage added to the anxiety. Several specific issues were related to feeling cold in air conditioning; the possibility of house fires as a result of faulty air conditioning, fears about having to go into a nursing home; and the cost of using air conditioning. Persons with congestive heart failure were often afraid to drink extra fluids due to medical advice about restricting fluid intake. Often mentioned was that security fears underpinned the reluctance by some older persons to open windows and doors. Furthermore, a fear of falling during the night and not being found was also identified as being a barrier to fluid intake:

“*I think a lot of people don’t drink enough because they don’t want to have to get up in the middle of the night to go to the toilet because they’re afraid they’re going to fall.*”

(Executive Officer, Country Hospital)

Notably, barriers to seeking assistance during the heat included older persons not wanting to be a bother to others, the thought of spending hours in hospital corridors, and apprehensions about being perceived as being unable to cope. A loss of independence as a consequence was seen as a major fear:

“*A particular one I came across was, the lady wouldn’t ask for help because that would then give the family cause to put her into a nursing home because … they feel she wouldn’t be able to cope. That was her fear.*”

(Social worker, Government Department)

### 3.4. Adaptive Strategies

Adaptive strategies were split into enablers and barriers (Table 2). Enablers included adaptive behaviors, cooling centers, neighbor and family support, and resilience. Barriers to adaptation were more numerous however, covering a range of issues which often intersected with those previously identified as being physiological, psychological or socioeconomic.

Stakeholders spoke of some older clients having an aversion to air conditioning because of a dislike of cold air blowing on them, some believing it will make them ill. Additionally, some feel the cold more than the heat, particularly if they are sedentary or have muscle wastage, and prefer to be warm rather than cool. 

Several stakeholders from different professional backgrounds mentioned that the use of modern reverse cycle air conditioners can be troublesome for some older people. Unfamiliarity with complex operating instructions together with small heat/cool symbols that are hard to see and decipher, can lead to confusion:

“*Some people can’t read the words, some people can’t see the icons and aren’t used to icons. And they put them on heat instead of cool*.”

(Senior policymaker, Government Department)

“*The actual changing over of the air-conditioner from heating cycle to cooling cycle is sometimes very confusing for elderly folk. And we have actually found numbers of our clients, who when the (…) have gone in, have found that the air-conditioners have been blowing hot air, not cold air. They haven’t known to switch it over.*”

(Manager, Non-Government Organization)

For people with dementia, adaptation can be hampered by a range of multifactorial physiological and psychological issues that can affect behavior. Another issue raised by two respondents was that in later life elderly migrants often revert to their first language, which can increase isolation.

It was identified that some older people have fixed opinions, and are defiant or reluctant to change behaviors during extreme heat, and it was thought many lacked insight into the potential dangers of excessive heat exposure. It was recognised that life experience may have played a role in shaping some of these attitudes whilst also contributing to resilience. Referring to older people in South Australia’s warm climate, an emergency services manager said that having lived through the war era, the Great Depression and the years prior to electrical appliances, “in some ways they’re less vulnerable,” as “they’ve got techniques” of keeping cool “that are drummed into them from an early age”. Another respondent said:

“*And I think a lot of elderly people have that kind of attitude you know, they’re very stubborn, very proud of being able to look after themselves. And I think that’s fair enough too. They’re people who have looked after themselves all their lives and probably gone through much harder times than we’ve gone through*.”

(Policymaker, Government Department)

A social worker reported that during power failures her elderly clients often coped better than younger persons with neurological conditions, perhaps due to heat preservation techniques learnt in pre-air conditioning times. It was said that the vast majority can cope and implying they are unable to cope may be offensive to those who value their autonomy. Narratives revealed that negative attitudes to change may also be grounded in resilience and a resistance to embrace anything not a part of the generational culture. It was noted however, that this attitude may not be appropriate for aging persons:

“*Certainly there’s a lot of that generational side to it, that this is what we do, we tough it out, and it should be exactly the same. They haven’t taken into consideration that they’ve changed in that time and that their body might not be so well adapted to … coping with the heat anymore*.”

(Director, Health Services)

Stakeholders recalled experiences of the severe 2009 heatwave which subsequently prompted the introduction of government policies and heatwave plans. It was noted that for some seniors, an air conditioned hospital provided a refuge from the heat during the heatwave. One local government officer recalled desperate cases of feigned illness to facilitate hospitalization, and a health services director cited instances of patients’ delayed discharge from hospital if in-home air conditioning could not be guaranteed. Managers spoke of heat-relief or cooling centers being setting up in country hospitals and residential care facilities, where older people could sit in a comfortable, air conditioned environment. This was seen as an effective primary health care strategy. Not wanting to leave animals was said to be a contributing factor to these centers being under-utilized, and consequently one executive was willing to allow small pets at the facility in order to overcome this barrier. Other reasons cited for people not going to publicly air conditioned spaces included: limited access to transport, financial constraints, mobility issues, and reluctance to leave the comfort and safety of their home. Opinions about promoting publicly available cooling centers such as shopping malls were mixed amongst the stakeholders. The logistics of the elderly traveling in the heat to cooling centers and requiring nourishment (and possibly extra care) whilst in the centre, was viewed by some as being problematic. One policymaker spoke strongly against the concept, saying it was much better to encourage resilience and support people “in their own environment”.

Interruptions to power supplies were noted to be a concern during extreme heat and aged care workers were mindful of the need to carefully manage their residents in the event of power failures. The problems of electricity dependent cordless telephones and the benefits of also having a traditional landline were mentioned. Being prepared for the consequences of power failures was seen as an important resilience strategy. Additionally, stakeholders indicated that older persons were often concerned that overuse of air conditioners could jeopardize power supplies during heatwaves and would therefore limit their use:

“*…and we have to say, no hold on, you put yours on. The 20 year olds next door they can cope with a bit of heat, but, you can’t.*”

(Manager, Non-Government Organization)

## 4. Discussion

This study was undertaken the year following an extreme heatwave during which some 690 people attended hospitals with heat-related illnesses [19]. Heat-related emergency department presentations were greatest in those aged 75+ years [11]. Stakeholders who took part in this study included key informants from local and state government, emergency services, hospitals, the aged care sector, and organizations/services dealing with aged clients. As well as reinforcing existing knowledge in geriatric heat-health, this qualitative study has identified some new insights into risk factors, and findings are well placed to inform a large telephone survey to explore perceptions, beliefs and behaviors of the elderly population during heat episodes. 

From discussions with stakeholders it emerged that heat-susceptibility in the aged can be affected by broadly defined issues relating to: physiological, socioeconomic and psychological factors, and enablers and barriers to adaptation. These issues often overlap with a lack of clear delineation between specific risk factors. 

Narratives identified physiological issues including poor health, chronic conditions and functional disabilities as important in underpinning the heat-susceptibility of older people, as noted by others [7]. Additionally, as reported elsewhere, dehydration in the elderly is common, particularly during hot weather [20], being due in part to a reduced thirst sensation with age [21]. Maintaining fluid balance can be problematic as the incidence of not only dehydration, but also hyponatremia has been shown to increase during heatwaves [22]. 

Our findings showed that socioeconomic issues were very influential in shaping behaviors. Whilst approximately 85% of South Australian homes have a cooler [23], usage can be associated with household income [24]. Concerns related to power costs figured prominently in discussions regarding the use of air conditioning, as noted in other studies [25]. Publicly cooled spaces such as shopping centers can provide a cool environment without the worry of personal power costs, but can be problematic due to the lack of care facilities [26] and transport to and from the centers in the heat. A unique and practical alternative raised in this study was the establishment of heat-relief centers in designated areas of small hospitals and residential care facilities. This option, which overcomes some of the logistical problems associated with for example, shopping centers, may also help reduce unnecessary or extended hospitalizations of the vulnerable elderly.

The incorrect use of air conditioners was an interesting point raised by several respondents. The confusion for some older persons who had mistakenly had their systems set to ‘heat’ may be due to cognitive impairments, a general unawareness of the need to alter settings at the change of season, or lack of knowledge of how to do so. Small buttons and hard to read symbols on modern air conditioners are design features that could be modified to be more user-friendly for the frail elderly. A recent study of comfort controls in Californian homes also identified problems with modern thermostats including controls and settings that are difficult to understand or change, and symbols that are inconsistent between models [27]. An ‘automatic’ setting which is a feature of newer air conditioners, avoids the need to alter the mode at the beginning of summer, and should be pointed out to householders during installation. 

Our study has highlighted anxieties that heat can exacerbate in older persons, indicating a psychological aspect that has been rarely described in the literature. Whilst a significant effect of heatwaves on mental health has been previously reported in Adelaide [28], the breadth of anxieties directly or indirectly associated with extreme heat may have been underestimated to date. The link between psychological and physical heat-health therefore warrants further investigation both in Australia and elsewhere.

Contextualizing the issues which underpin heat-susceptibility in older populations requires an understanding of contributing factors throughout the life course, as was recognised by some respondents. Underlying cultural, institutional, and infrastructural factors should be considered [29] as well as social, economic and political factors [2]. The Australian Bureau of Statistics defines persons born before 1927 as being of the ‘oldest generation’ and those born between 1926 and 1946 as the ‘lucky generation’ [30]. It is the ‘oldest’ generation who are likely to be the most vulnerable to heat due not only to their age and declining health status, but also other factors. These persons have lived through the Great Depression and world wars, and many have limited formal education. Approximately one third of this generation live alone and of these, 4 out of 5 are widowed [30], which in itself is a contributing factor to heat-susceptibility [3]. A considerable proportion of the summers experienced by these people have been pre-air conditioning, and many may now be unable to engage in proactive measures [2] due to their advanced age. Notwithstanding, acclimatization and a lifetime of experience with hot summers can clearly reduce vulnerability, particularly in the healthy. Understandably, many older people consider themselves well in control and able to cope, and suggestions otherwise may be offensive. This comparative optimism, when one’s perception of risk of a health crisis is lower than that of a similar other’s, has been noted previously in older adults who feel they have a strong sense of control [31].

Those of the ‘lucky generation’, currently aged 65 to 84 years [30] and to an even greater extent, the following ‘baby boomer’ generation, may not have the cultural constraints of their forebears, and may be more likely to use cooling devices. More than one third of this generation was born overseas, many immigrating to Australia from Europe in the 1950s and 1960s. An important point raised in this study is that linguistic barriers can emerge for some aging immigrants if first language reversion occurs. This phenomenon, where use of the second language recedes and the first language increases [32], could be a significant issue during heatwaves that may affect the uptake of heat-health information in increasingly multicultural societies.

Although heatwave plans are now common in Australia [13,33,34,35] and other countries, the incidence of heat stress in the elderly may increase in coming years for several reasons. Firstly, it is widely accepted that climate change is contributing to rising temperatures and the likelihood of more frequent, intense and extended heatwaves [14], and even in acclimatized populations there are limits to heat tolerance [36]. Secondly, despite the potential for forthcoming elderly generations to be more heat-adaptive than their forebears, demographic changes mean there will be more persons at risk. By 2051, it is predicted more than one in four Australians will be aged 65 years or over [37] and the prevalence of dementia will increase substantially [38]. As in previous studies [28], dementia sufferers were highlighted as being a group at risk in this study. Finally, the generations born since the latter half of the 20^th^ century have for the most part not experienced summers without air conditioning, and in the event of power failures, may be less resilient and unfamiliar with alternative proactive measures to avoid heat stress. Indeed, it has been reported that during the 2009 Adelaide heatwave, mortality was greater in the 15–64 year age group than the elderly [11]. Factors such as adaptation to heatwaves, good care for the elderly, and social cohesion in this age group are likely contributing factors [39]. Younger persons would therefore benefit from familiarization with non energy-intensive heat-adaptive behaviors (soft adaptation) rather than relying solely on air conditioning (hard adaptation), in order to build resilience during heatwaves and associated power failures. 

The main limitation of this study is that the findings stem from experiential knowledge of stakeholders in South Australia only, and that other barriers to adaptation may exist in more vulnerable and less acclimatized older populations elsewhere. Thematic analysis is based on subjective interpretation of the data and themes identified may vary between researchers. This study has nevertheless addressed the question ‘Why are older people vulnerable during hot weather?’, and has identified important aspects of heat susceptibility that may not be captured in other study designs, whilst also informing a quantitative survey. Further mixed methods research is warranted to identify those most at-risk in the community.

## 5. Conclusions

Behavioral adaptations during extreme heat are necessary for older persons whose thermoregulatory abilities may be compromised due to age or disease. This investigation of age-related heat-susceptibility and barriers to adaptation has highlighted several issues which may potentially be addressed with public health interventions or policies. Although some findings resonate with similar studies elsewhere, other lesser known issues have emerged that may affect the adaptive capacity of older persons during heat waves both in Australia and elsewhere. 

It is important that operating instructions for reverse cycle air conditioners be made clear to householders at installation, and user friendly designs conducive to use by those with impaired vision should be considered. It is also strongly recommended that carers and family members of the aged check air conditioner settings as summer approaches. 

In recognition of the economic disadvantage experienced by some seniors, rebates for energy costs to the most vulnerable may help alleviate financial concerns about air conditioner usage during summer. Overcoming this barrier may be a cost benefit to the health sector if adverse outcomes are averted.

When providing advice to keep hydrated during hot spells, authorities should also be cognizant of persons with certain medical conditions and those taking medications that can interfere with thermoregulation. More research needs to be undertaken in this area to inform guidelines regarding the management of certain conditions during extreme weather. 

Stakeholders participating in this study said they felt the research was of value to their organization. By conducting interviews and focus groups, we were able to synthesize a higher level collective knowledge, which has shed light on the underlying reasons why some older people fail to change behaviors and/or become ill during heatwaves. This provides policymakers, emergency services, decision makers and health professionals with information upon which to base future heat management plans for seniors. Furthermore, this study has informed a survey instrument for use in the second stage of the research, enabling triangulation of qualitative and quantitative data to comprehensively address the issue of vulnerability to heat in older populations. 

Promoting adaptive behaviors and improving health literacy will undoubtedly help reduce heat-related morbidity and mortality in the elderly. With the progression of climate change and the likely occurrence of unprecedented heat events, preventive measures along with primary health care initiatives will continue to be important public health strategies to help overcome barriers to adaptation during heatwaves. 
